# Assessment of anorexia nervosa: an overview of universal issues and contextual challenges

**DOI:** 10.1186/2050-2974-1-29

**Published:** 2013-08-09

**Authors:** Lois J Surgenor, Sarah Maguire

**Affiliations:** 1 Department of Psychological Medicine, University of Otago at Christchurch, 4 Oxford Terrace, Christchurch 8140, New Zealand; 2 Centre for Eating and Dieting Disorders, University of Sydney, 92-94 Parramatta Road, Camberdown, New South Wales 2050, Australia

**Keywords:** Anorexia nervosa, Assessment, Eating disorders

## Abstract

**Aim:**

Anorexia Nervosa (AN) is a complex and clinically challenging syndrome. Intended for specialist audiences, this narrative review aims to summarise the available literature related to assessment in the adult patient context, synthesising both research evidence and clinical consensus guidelines.

**Method:**

We provide a review of the available literature on specialist assessment of AN focusing on common trajectories into assessment, obstacles accessing assessment, common presenting issues and barriers to the assessment process, the necessary scope of assessment, and tools and techniques. It describes the further step of synthesising assessment information in ways that can inform resultant care plans.

**Results:**

In addition to assessment of core behaviours and diagnostic skills, considerations for the expert assessor include the functions of primary care, systemic and personal barriers, knowledge of current assessment tools and research pertaining to comorbid pathology in AN, assessing severity of illness, role of family at assessment, as well as medical, nutritional and compulsory elements of assessment.

**Conclusion:**

Comprehensive assessment of AN in the current healthcare context still remains largely the remit of the specialist ED clinician. Assessment should remain an on-going process, paying particular attention to available empirical evidence, thereby reducing the gap between research and practice.

## Introduction

Most health workers recognise that anorexia nervosa (AN) is a serious disorder that results in significant disability and impaired quality of life. Unfortunately for some, this disorder becomes a chronic or life-shortening illness. It is well-established that AN has the highest mortality rate of all psychiatric disorders [1]; compared with their peers without the illness the risk of premature death is approximately ten-fold in a person with AN [2]. Health consequences are widespread, both in terms of direct physical and mental health effects and economic costs to society overall; a recent Australian analysis placed the total yearly direct health system costs of AN at $59.8 million [3]. This means that although AN and AN-like conditions are relatively rare, severe and enduring forms of AN have impairment outcomes comparable to people with moderately severe depression and schizophrenia [3].

Assessment and a resulting early diagnosis become a vital task for the health system and current research continues to suggest that, although primary care is the context of most presentation [4] areas with specialist services have more than double the number of diagnosable eating disorder (ED) cases, suggesting a sizeable proportion of diagnoses do not occur until the specialist service level [5]. What this means for both settings is worth discussion, but regardless, assessment skills and knowledge of the evidence base pertaining to diagnoses remain an important part of the skill set of the specialist clinician.

Regardless of the setting and complexity, there are descriptions of ‘ideal practice’ in the assessment of the disorder and associated health difficulties. However, assessment approaches do vary, along with models of service delivery, admission thresholds and the resources available in specialist settings [5]. Such factors will influence the composition of a multidisciplinary assessment team. There are suggestions about what constitutes an ‘ideal’ assessment team, but rarely will services have all recommended components, and multiple tasks may fall to ‘mini teams’ [6]. There is however areas of general consensus about many necessary and desirable tasks of assessment and given the above constraints it often falls to the specialist clinician working in relative isolation to be well versed in all factors pertinent to a comprehensive assessment. Focusing primarily on adult populations, this paper aims to reduce the gap between research and practice (as it relates to assessment of AN) by firstly providing a comprehensive review of the current literature relating to assessment pathways, barriers to assessment, diagnostic issues, instruments for assessment of illness and severity, role of the family at assessment, comorbid presentations relevant to assessment, medical, nutritional and compulsory assessment practises. Secondly, it discusses the process of synthesising information gathered at assessment for the purposes of formulation and ultimately to direct treatment.

### Pathways to assessment

#### Links with primary care providers

Given the scarcity of specialist ED services, most people with AN will not be seen by a specialist service in the first instance, and for a significant number, specialist assessment may not occur at any stage. Half of those with an ED are first diagnosed by their primary care physician, although of concern a sizeable minority may go through life without any help whether this is for AN or other emotional problems [4]. General practitioners (GPs) and other primary health care providers are best placed to be the health practitioners first involved in conducting preliminary assessments, providing initial triage, and thereafter sharing case management with other clinicians [7]. This is partly because people with eating disorders attend GPs and other medical speciality services more frequently than their peer group, albeit often for conditions seemingly unrelated to the disorder. The presence of an ED also increases the rate of presentations to Emergency Departments [4]. In terms of accuracy of diagnosis, AN is the ED most often accurately diagnosed by primary care physicians [8]. Atypical cases, or those who do not present with all diagnostic criteria, can be misdiagnosed [9] or considered ‘less serious’ despite clearly having a clinically significant disorder that squarely sits within the AN spectrum.

A number of obstacles arise for primary care health professionals in their attempt to detect AN and refer on to ED services. Setting obstacles include the limited consultation time available and relatively limited exposure to AN. Clinical obstacles include patient minimisation of behavioural and psychological symptoms, the diversity of symptom expression, and well-hidden symptoms [7]. Further, there may be difficulty in determining which symptoms are most important in assessing medical acuity or how severe the condition has become.

Systemic issues also contribute to rates of diagnosis and their accuracy. GP education about risk factors and early warning signs as well as screening instruments like the SCOFF [10] can assist detection and management, particularly where GPs are expected to coordinate and manage concurrent medical and psychiatric conditions. Close and regular liaison with ED specialists may encourage more regular enquiry about eating difficulties in those who frequently present with emotional and/or physical problems. GPs are referral “gatekeepers” (whether intended or otherwise), and ED clinicians have a role in supporting and educating their colleagues about frontline assessment practices and referral thresholds. In the regions where there are specialist services, GPs are likely to have greater awareness of eating disorders and in turn refer to specialist services more frequently [6]. In any event, presentation to primary care or emergency services should always be used as an opportunity to introduce, or re-engage with an ED clinician or ED service if one is available.

#### Facilitating assessment following referral

Early identification and treatment of AN is consistently argued as a means to reduce the duration of AN [11,12]. A systematic review of treatment seeking has recently estimated that the median delay from onset to treatment for AN is 15 years [13]. Identifying the barriers between these two time points becomes pivotal, and will likely vary. A significant minority of people referred for ED problems fail to attend an initial assessment [14], and further significant attrition occurs between assessment and providing and/or completing treatment. The following sections discuss common obstacles and possible solutions.

#### System and resource barriers

There are extensive system factors contributing to delays in accessing assessment. The shortage of services often results in prolonged waiting lists for assessment, meaning that by the time of assessment, patients may be demotivated or otherwise less likely to engage in what is offered. Tatham et al. [15] trialled an active ‘opt in’ waitlist management strategy for an ED clinic, whereby following initial assessment patients were required to actively select to remain on the waitlist for treatment. While ‘opting in’ letters may reduce the waiting time for an assessment [16], this triage approach is not without significant risk. Specifically, those most in need of assessment may be the least able or willing to ‘opt in’.

Long assessment waiting lists are likely to pose a barrier to engaging in any subsequent assessment - just as these do in many other health settings. It has been reported by patients that this can send a distorted message that the disorder is not serious enough. World-wide, services are caught in the ethical dilemma of setting priorities for how scarce resources should be allocated and prioritised. As these decisions have major clinical implications, resource priority-setting should be reviewed at regular intervals. Suggested strategies that have been shown to increase the likelihood of a valid referral resulting in an assessment include direct phone contact with the patient [14] accurate written knowledge services and, where appropriate, steps to engage significant others (for example, family) in the assessment process [11].

#### Barriers of ambivalence and motivation

There are multiple psychological reasons why people with AN may be reluctant to present for assessment or are guarded during an assessment. The disorder itself, at best, presents genuine ambiguity for many who suffer from it. As one woman described it, “I want to get rid of the disorder but not the body shape (of AN)…I want the best of both worlds” [17], page 29. AN has been demonstrated to serve more pivotal functions of affect numbing or identity and the individual may see no reason to alter it at all or, as is most common, the patient may move between wanting to address it and not seeing it as a problem. This ambivalence is complex and may shift even in the preliminary assessment interview. The interview may be infused with ‘bargaining points’ or requests for ‘conditional’ or no treatment at all. With these dynamics at play, careful clinical skills are required. It is important that such bargaining is not seen as ‘manipulation’ with the negative connotations of that concept. Rather, it represents a genuine struggle with symptoms that are both controlling and out of control, and at times deeply confusing for the person. It can be very validating to the client, and move the process of assessment along smoothly, to express understanding for the two seemingly opposite positions that can be held within the one person, and to not dismiss or invalidate the part of the client that wants to retain the illness. Family and carers may be equally conflicted about the need for treatment, with parents sometimes caught between loyalty for their offspring and wanting to heed professional advice. Assessment of family functioning is discussed later in this paper.

Although overlapping with ambivalence, motivation is a wider construct involving cognitive, behavioural, and biological systems. Appraising motivation for treatment and ability to change is an important component of any initial assessment: it often contributes to case formulation, decisions made at the conclusion of assessment, and subsequent treatment planning. Yet assessment of this is far from straight forward. Motivation may be symptom-specific: there may be high engagement in strategies to control physical side effects, but minimal engagement in behaviours directed towards weight gain. Engaging in conversations that highlight this variability at the beginning of a therapeutic interaction is likely to build alliance. As noted by [18], assessing what makes people want to recover, and what recovery represents to them, is important, and this is something highly valued by patients themselves.

Of note, there is controversy regarding the ability of clinicians to accurately assess motivation, and whether this predicts anything about the likelihood of engagement or even treatment outcome. Waller [19] provides a compelling overview of the complexities involved, including clinician overreliance on verbal expressions of change. Interestingly, similar critical analyses are now being extended to motivational enhancement techniques, noting that in regard to AN, the evidence is mixed at best [20]. Rieger et al. [21] specifically suggests motivation is not only required to alter the behavioural aspects of the illness (namely how much weight are patients are prepared to gain each week until they reach a healthy weight, and how motivated they feel to be at a normal weight) but also feeling of self-efficacy about achieving this. Some patients will state that they are very motivated to be at a normal weight and would be prepared to gain up to a kilo a week to do so, but when asked how confident they are that they can actually do this, the answer is quite different.

#### Clinicians as barriers

People presenting for assessment are often fearful of being judged or criticised. These fears are not unfounded as it has been repeatedly demonstrated that attitudes of health professionals, including mental health specialists towards EDs, are not always positive [22], and are no more empathic than those of non-professionals [23]. Negative professional attitudes may arise through lack of training or experience [24], inadequate resources and work pressure, and genuinely held stereotypes about AN being a personal choice. These factors can contribute to AN being seen as a disorder with relatively low prestige by health care professionals [25]. Training and support strategies may go some way to counter these effects. As clinical experience decreases the likelihood of negative reactions, services significantly benefit from retaining highly skilled staff.

### Early drop-out

Failure to engage, or drop out once treatment begins has a negative association with prognosis [26,27] and increases the likelihood of intensive treatment utilisation in later stages of illness [28]. Specialist services have lower treatment drop out and allow greater continuity of care with the same service, yet studies suggest that amongst those with EDs who actually attend assessment, approximately one-in-five drop out before treatment can start [29]. Reliable predictors (clinical or otherwise) of drop-out from services are scarce [17,26,30], meaning that future assessment interviews should be seen as an opportunity to explore the factors involved from the patient’s perspective. Hudson et al. [31] found life-time treatment for AN to be 33.8%, and thus much lower than other DSM-IV eating disorders (BN 43.2%; BED 43.6). Reducing drop-out before and after assessment is an obvious means to increase treatment take-up.

### Diagnostic issues at assessment

AN is a syndrome involving considerable symptom variability between and within cases over time. Cross-over between AN subtypes is common, suggesting that those with AN-Restricting Type and AN-Binge-Eating/Purging Type may be phases of the same condition rather than distinct groups [32]. Diagnostic weight cut-offs are debated [9] and the severity of psychological versus physical symptomatology may not always highly correlate, albeit these will be highly intertwined at extremely low weights due to the behavioural, psychological, and cognitive effects of starvation [33]. The following sections discuss key issues regarding diagnostic variability.

#### Revised diagnostic criteria

The American Psychiatric Association diagnostic criteria (DSM-5) [34] have recently been reviewed. Hebebrand et al. [9] comprehensively discusses the rationale behind revised criteria, highlighting the reframing of psychological symptoms so that some pejorative attitudes are removed and other criteria are redefined to reflect the evidence. For example, eliminating the term ‘refusal’ (to maintain body weight), and replacing this with more objective terms describing food restriction. The removal of the diagnostic criteria of amenorrhea in post-menarcheal females is well overdue given the mounting evidence that this is of questionable utility. Similar changes are proposed for ICD-11 [35]. Clinicians involved in the assessment of AN should become familiar with current and future diagnostic debates in order to reduce the risk of adopting a rigid or outdated approach to formulating core psychopathology of AN and the expressions of this.

#### Effect of symptoms

Some features of AN can be difficult to assess meaningfully in that they decrease in ‘severity’ as illness severity increases. For example, fear of fatness can decrease as the individual’s weight drops and they are no longer confronted with fat on their own body. Similarly, drive for thinness and body image dissatisfaction can also decrease as weight decreases. To generalist clinicians, this could result in a failure to diagnose individuals with potentially severe AN, as they appear to lack either one or both of the essential symptoms. Other clinicians may misconstrue this as ‘denial’ and engage in confrontational discussions with patients, which may be unhelpful.

At very low weights, the effects of starvation will always distort the expression of distress, and patients in general are not necessarily aware of why they behave in certain ways or why they may pursue certain goals. It may be useful to assess certain symptoms from the perspective of what it would be like not to have them. For example, assessing body dissatisfaction/drive for thinness not from the standpoint of how the person with AN feels about their body at its current weight, but rather how they would feel about their body, and how intense their drive to lose weight would be if they imagined their weight being within a normal weight range, holds some utility [36].

#### Culture and ethnicity

AN may present differently in people with a non-Western background (see Soh et al. [37] for a review), and there are significant body composition differences across ethnicities. For example, Asian women show higher levels of body fat for the same BMI than their Western peers [38]. There is more mixed evidence that cultural issues influence psychological symptom variability. While a commonly cited difference is that fear of fatness is less evident in patients from a non-Western background, many studies do not show this [39]. Likewise, levels of body image disturbance amongst clinical groups from different cultures have been found to be largely identical despite genuine cultural differences in body image concern in those who do not have an ED [40]. Studies that have historically highlighted symptom variability across cultures have commonly examined ethnic minorities residing in Western countries: when an ethnic group is studied in their country of origin, many of the proposed differences in clinical presentation cannot be found. Likewise, struggles with psychological control reported in AN appear universal, but importantly it is deviation from the cultural norm (whatever the culture) that seems to distinguish women with EDs from their peer group [41]. In short, rather than ethnicity explaining any noted symptom variations, the pertinent assessment issues may relate to understanding the current cultural norms and their dissonance with the culture of their ethnic background.

#### Gender

Although representing around one in ten people with AN [42], males with this condition are much less likely to be recognised. Amongst those who do present for assessment, medical complications are more likely apparent than with their female counterparts [43]. There are mixed views about whether males present for assessment much earlier (see Carlat et al. [44]) or later (see Siegel et al. [43]) than their female counterparts. Psychological symptoms are largely identical, although there is some limited evidence that males may have less severe scores on diagnostic tools and symptom rating scales [45] and are more likely present with particular weight control strategies (for example, excessive exercise [46]) and dissatisfaction with muscle size and shape. Too few studies have been conducted to examine whether the natural course of the illness differs by gender. Some studies report a more benign outcome in men at one year (e.g. Strober et al. [47]), while a recent long term follow-up study found no gender differences in 10-year survival rates [48]. Causal models particularly relevant to males are still largely speculative, although sexual orientation issues feature prominently in males [49]. However it may be that gay men may be more willing to seek help for psychological problems. At this stage therefore, the assessment of males presenting with AN largely should follow the same assessment considerations as females.

### Assessment screening and severity tools

#### Screening tools

A large number of standardised self-report and interview-based measures are available to clinicians. There is longstanding debate about the superiority of interviews as opposed to self-report measures (whether paper and pencil or computerised), and ultimately the selection of any measure will depend on the purpose and context of assessment, along with constraints on time, availability of training and clinical factors. These issues are now discussed.

Self-report questionnaires are easy and quick to administer, although they differ in their utility. Shorter measures such as the Eating Attitudes Test (EAT-12; EAT-26) [50] and SCOFF [10] are useful in primary care situations where an ED may be suspected (NICE Guidelines; Allen et al., 2011), and identifying the need for second screening steps is a focus. However these are less useful in specialist settings where the more relevant task is to gather systematic information about severity and the extent of psychopathology. Here, longer self-report measures (for example, Eating Disorder Inventory (EDI-3) [51], Eating Disorder Examination Questionnaire (EDE-Q) [52], and Yale-Brown-Cornell Eating Disorders Scale (YBC-EDS, [53]) tend to assess two factors - a combination of risk factors and psychological disturbances associated with AN [54]. Detailed information about psychological symptoms can be elicited from these, which in turn adds to any clinical formulations. As standardised measures, they can be useful in assessing symptom change.

Semi-structured or structured clinical interviews such as the Structured Clinical Interview for DSM-IV Axis I Disorders Clinical Version (SCID-CV) [55], and Eating Disorder Examination (EDE) [56] are often referred to as “*gold standards*” (APA, 2006, p.46). These are more time-consuming, involve training and are more likely suitable for research purposes rather than clinical settings, although they do possess the advantage of being validated to render an ED diagnosis.

Irrespective of the measure used, scores are affected by a number of clinical, demographic and psychometric issues. Denial may occur in interviews due to embarrassment and reluctance to disclose, whereas the risk with self-report measures is that patients may inaccurately estimate the seriousness of core symptoms [57,58], or simply disagree that symptoms are problematic [59]. In very severe cases, administration of psychometric tests (AN-related or otherwise) is clearly inappropriate. Wider psychometric limitations arise from differences in international norms and few national clinical norms [54], and the utility of measures differ between adults and adolescents [60]. Furthermore, different formats of the same measure (e.g. EDE and EDE-Q) have only modest agreement ratings, with rates of agreement being dependent on the type of symptom being assessed [58]. In terms of AN, no measure reliably distinguishes between AN and EDNOS-AN [61].

A positive diagnosis can be made in most cases without the need for a battery of psychometric tests. The more useful place of standardised psychometric tools is to screen, assess symptom change and research. In the end, standardised psychometric tools cannot replace the advantages of a thorough clinical interview. As noted by Nordbø et al. [62], “*an assessment tool should never be regarded as a substitute for treating the patients individually or replace the need for an individual exploration in each single patient*” (page 660). This is further discussed in the final section of this paper.

#### What constitutes severity?

The use of the concept of illness severity is common in ED settings, and there are solid grounds for this. For example, severity markers can help aid treatment decisions, potentially aid prognosis, and promote more uniform international understandings about clinical presentations [63]. However, empirically supported definitions of severity are largely lacking. Clinicians commonly default to one or more symptom markers or treatment intensity markers such as BMI [64] or multiple severity indices based on different symptoms (not all of which are AN symptoms [65]), hospitalisation [66] or length of illness [67]. In an attempt to address problems with all of the above strategies, a more recent approach has been the development of empirically derived tools to stage AN severity [36,68]. Early indicators suggest that such measures involve multiple symptom dimensions, and neatly capture both clinical reality and patient subjective reality [36]. One of these instruments [67], the CASIAN, largely assess the behavioural features of the ED so as to minimise the factors of denial and the often perverse reduction in psychological distress discussed above, as the illness progresses (i.e. drive for thinness and body dissatisfaction can decrease with severity). Longitudinal data is still needed to determine how staging instruments could usefully aid assessment, treatment pathway decisions and the prediction of prognosis.

### Family involvement and family assessment

Where possible, it is highly desirable to have carers or family involved in the assessment process given they provide much of the on-going care and share the extensive burden of the disorder. For younger patients still living at home, family members may need to be directly involved as treatment agents [69] as long as they are appropriately supported and advised, noting that family members can become entangled with behaviours that maintain the disorder [70]. For adolescents, the assessment phase may more directly involve assessment of family functioning. The inevitable distress about having a family member with AN may need addressing in its own right and assessment of family functioning is core where family therapy treatment models are to be employed. There is no clearly superior way to assess family functioning: approaches are likely to reflect the model of family therapy employed and there are several variants of this. Family therapy models have been met with enthusiasm although a recent Cochrane Collaboration review suggests a need to temper this enthusiasm due to a range of methodological shortcomings in the small number of published treatment trials [71]. Such moderation should equally apply to assessment processes meaning that rigid approaches to assess families should be avoided.

### Co-morbidity

Given psychiatric co-morbidity is common in people with AN, consideration of these associated problems should be routine. Those presenting for assessment may have higher rates of co-morbidity than AN community samples due to the additional burden and distress of these comorbid conditions. Indeed, these conditions may be the precipitant for presentation rather than AN itself. If psychiatric co-morbidity is recognised, conclusions about the actual presence of a primary anxiety (or mood disorder) is often best delayed until the symptom contribution of AN can be clarified, especially in the case of severe AN. Co-morbidity may impact on treatment engagement, in both directions, with co-morbidity prompting assessment or in other circumstances reducing engagement. The following sections discuss common forms of co-morbidity and implications for assessment.

#### Mood disorders

While major depression features prominently, depressive symptoms may also result from severe malnutrition, which almost certainly elevate these rates. Generally, however, there is robust evidence for elevated rates of major depression in all AN populations, irrespective of whether these are clinic or community samples. Godart et al. [72] found life-time prevalence rates ranging from 9.5% - 71.3%, with the variability in findings likely due to methodological differences across studies. The overall lifetime prevalence for eating disorders patients is estimated at 40%. There is debate about the most common sequencing of these comorbidities with some suggesting that the mood disorder more often precedes the ED (Cooper et al., 2002) and others highlighting past problems of over-diagnosing depression when instead symptoms may be secondary to malnutrition [57]. The consensus is that the relationship is complex. Scores on depression screening measures can be significantly elevated by an ED [73] resulting in a need to set higher cut-off scores in order to be meaningful. Vigilance for mood symptoms should also be ongoing given that improvements in AN can be experienced as depressogenic, with some sufferers construing the relinquishing of symptoms as a sign of losing control or giving in (to treatment) [74].

Bipolar disorder co-morbidity in AN is rare [75]. Affective disorders along this spectrum are more likely to be associated with forms of disordered eating involving bingeing or over-eating. How EDs and bipolar disorders overlap requires further study.

#### Assessment of self-harm and suicide risk

A recent meta-analysis observed a decreased risk of completed suicide in AN [76] over recent decades, with a standardised mortality ratio (SMR) of 31. This SMR still represents an elevated risk when compared with other EDs and the general population. The extent to which AN directly contributes to this risk as opposed to the combined effects of AN and co-morbidity to this risk is still debated [77].

Information gathering about risk comprises a two stepped approach. First, standard suicide self-harm risk assessment considerations apply. Thus, just with non-ED populations, the presence of depression increases the risk of suicidal behaviours and completed suicide and a sizeable proportion of these people will have had contact with their GP in the week or months before death. Other risk factors include a history of mental disorder, more severe psychopathology overall, Axis 2 psychopathology, history of self-harm/suicide attempts, and substance misuse (see Hawton et al. [78]). It follows that assessment of these standard risk factors should always occur when engaging with a person presenting with AN.

Second, the presence of particular AN symptoms or a history of AN may produce additional risks. These include the presence of binge-purge patterns [79], markers of chronicity including illness duration and very low weight [80], and high anxiety in AN-R [79]. Rates of completed suicides in people with AN are debated [2], with one study reporting suicide as the second most frequent cause of death in a retrospective study of those treated in an ED service [81]. The most consistent finding is approximately 20% of deaths in AN are attributable to suicide [82]. It is self-evident that assessment of suicidal ideation and behaviours is important, especially in those presenting with co-morbidity.

Unusual forms of self-harm have been reported that are directly linked with efforts to maintain AN. For example, ingesting large quantities of salt [83], scratching [84] and blood-letting [85]. How these phenomena fall under the umbrella of wider deliberate self-harm (DSH) phenomena is unclear as DSH has multiple intended functions [86]. If present, such behaviours are unlikely to come to light in outpatient assessment settings. Although the conscientious clinician will assess for them, these may only be revealed following close monitoring, as occurs in inpatient settings.

#### Anxiety disorders

There is extensive overlap between anxiety and AN symptoms, with AN exacerbating concurrent anxiety disorders in addition to extensive anxiety symptoms forming part of the AN syndrome [87]. This makes assessment of anxiety a necessary component of any formulation, irrespective of any suspected Axis 1 anxiety disorder. This is because common anxiety treatment techniques inevitably will be part of treatment. Assessment questions include the extent of pre-morbid anxiety, the presence of genuine co-morbidity and the degree to which anxiety symptoms are actually part of AN. These issues are addressed in turn.

Reported lifetime prevalence rates of anxiety disorders vary considerably ranging from 23%-75% (see Swinbourne and Touyz [88] for a review). Generally the anxiety disorder is more likely to precede the onset of AN rather than the other way around [89], although this may depend on the anxiety disorder in question [90]. Of the anxiety disorders, obsessive-compulsive disorder (OCD) is the most researched condition [91], although social phobia, other phobic states and Generalised Anxiety Disorder (GAD) and anxiety-based problems such as clinical perfectionism [92] have all received attention.

Teasing out the functional relationship between wider anxiety phenomena and/or the presence of a primary anxiety disorder can be challenging, and even after weight-restoration anxiety symptoms tend to persist [93]. Detailed functional analysis of behavioural and cognitive rituals, the scope of intrusive thoughts, the scope of avoidance, and any other symptoms of fear may help clarify how these features are directly part of AN or more correctly attributed to another primary anxiety disorder. Importantly, anxiety symptoms may not be reported due to the success of avoidance or engaging in subtle “safety behaviours” that keep symptoms at bay. In such cases, anxiety symptoms may only emerge once there are treatment demands for change. As a result, the absence of anxiety symptoms on initial assessment should not be taken as evidence of the absence of anxiety.

Obsessions and compulsions particularly increase with starvation, and sometimes to an intensity that is comparable to people with OCD. Conclusions about the presence of a primary anxiety disorder should wait until the effects of starvation have abated.

#### Substance abuse and dependence

Reported rates of substance abuse in AN vary widely and are significantly associated with AN subtype (binge/purge), with rates as high as 35% in those who cross over to Bulimia Nervosa (BN) after weight restoration [94]. In general, the presence of any bulimic symptomatology significantly increases the likelihood of co-morbidity involving a variety of substances, both illicit and legal [95,96]. The sequencing of co-morbid alcohol problems and AN shows no distinctive pattern [97]. However the concurrent presence of alcohol misuse and AN should prompt enquiry into wider psychopathology given the increased odds of this in this subpopulation [98] and the known increased mortality [2].

Other substance abuse and dependence problems include the use of diet pills, laxatives, diuretics and other illicit stimulants for appetite suppression and increased metabolic effects [99]. Opioid and sedative use is also significantly elevated. In short, the assessment of substance misuse should cast a wide net, seeking information about illicit and over-the-counter drugs, including common substances containing high levels of caffeine. The assessment should also include patient understandings about how these substances maintain AN symptoms (for example, warding off normal signals of hunger) or manage other psychological symptoms or distress.

### Nutritional, weight, and eating behaviour assessment

Obtaining a weight and nutritional history is a critical component of assessment, although this can be far from straight-forward. Being asked to be weighed is one of the most anxiety-provoking demands for someone with AN, even when many know their weight due to excessive self-monitoring. In the situation of outright refusal to be weighed, this can be a temporary impasse if the anxiety about this is explored. Clinicians who allow the continued avoidance of assessing weight may find themselves inadvertently colluding with the disorder.

Body mass index (BMI) is the most common metric used, with extreme levels (BMI ≤ 13) consistently showing a risk of a poorer outcome and elevated medical risks [81,100]. Centile charts are recommended for patients who are younger than 18 years of age [101]. Percentage of body fat is another useful indicator of risk as this also has been associated with outcome [102].

Apart from ascertaining current weight, there are several aspects of weight history that are clinically important. These include obtaining information about highest and lowest-ever weight (BMI), means of weight control, and if amenorrhea is present, weight at which menses were lost or in the case of primary amenorrhea consideration of the effect of delayed puberty. A history of a very low BMI at any point in the illness trajectory is a risk factor for premature death [2]. BMI alone is problematic in younger populations for whom BMI-for-age is argued as more appropriate [103]. Patient perception of their ‘ideal’ weight, and how they determined this, can provide useful clinical information about maintaining factors, and how AN is serving to manage psychological difficulties. Commonly, a particular weight may be fixated upon with an expectation that achieving this will bring a sense of well-being and success. Inevitably such rewards are not forthcoming often resulting in a decision to work towards a lower weight - as if the original goal was miscalculated.

A food and fluid diary may help assess aspects of nutritional pathology, or at least start the discussion about nutritional intake. Shame, embarrassment, and anxiety about increasing nutritional intake, are amongst the factors that affect the accuracy of food diaries and other forms of self-recording. Additional enquiry should be made about avoided foods, behaviours and rituals around eating, evidence of self-imposed caloric limits, and the full repertoire of compensatory behaviours.

### Medical assessment

The main causes of increased mortality in AN are those directly related to the disease [82]. Fortunately advances in assessment and careful treatment of AN-related medical abnormalities have helped reduce the rates of mortality [104] compared with earlier practices. Multiple organ systems should be reviewed at assessment and abnormal medical findings can provide compelling objective evidence to ambivalent attendees who may contest the need for treatment. The types of medical complications presenting are influenced by age, clinical history, and weight history. The extent of medical and physical investigations undertaken will depend on these factors although there is some consensus on commonly recommended laboratory investigations. These include a full blood count, urea and electrolytes, biochemistry including calcium, magnesium, and phosphate, and glucose and liver functioning [105]. Assessment and monitoring for phosphate depletion is particularly important. Hypophosphatemia is well known to be associated with catastrophic consequences in the context of refeeding particularly for the severely underweight person.

Of concern, complications that are not reversible can occur in those who have yet to develop a chronic problem, and these include altered linear growth, osteoporosis, and structural or functional brain changes [106]. It is important to recognise that patients may initially appear asymptomatic due to slow adaptation to starvation over time. More detailed medical assessments may be indicated for those presenting with severe AN, as it is a mistake to consider mild AN as a benign condition. Even those with mild AN may experience prolonged periods of debilitating symptoms [11].

A full medical history and physical examination is essential to highlight immediate medical problems such as hypotension, hypothermia, bradycardia, skin conditions, and intercellular changes such as dehydration. Enquiry should extend to other gastrointestinal problems such as diarrhoea, constipation and abdominal cramping. A small number of patients may have had extensive previous medical work-up in an attempt to locate a medical condition that could explain symptoms that are more correctly caused by AN. Likewise, although rare, other non-psychiatric causes of significant weight loss (for example, medical conditions resulting in mal-absorbing consequences) should be considered early in the assessment process.

A medical review should include considering the presence of other conditions that may have serious consequences while occurring in the context of AN (e.g. diabetes) or other causes of weight loss unrelated to AN (e.g. primary gastrointestinal disorders), along with a review of medications that may compromise or contribute to medical risks occurring (for example, medications that prolong QT intervals). Since cardiac abnormalities have been estimated to occur in up to 86% of patients [107], and cardiovascular causes are a major cause of death [108], cardiac investigations (ECG) should check for a range of cardiomyopathies, of which there are many [109]. Studies suggest that is unclear when QT prolongation first occurs in the natural history of AN and when the period of greatest risk of cardiac abnormalities may occur [107]. Endocrine screens are useful, particularly checking thyroid functions and other hormonal disturbances.

Other checks may need to include bone density scans assessing for osteoporosis and spontaneous or low impact fractures, particularly if pain is a presenting complaint. When symptoms of self-induced vomiting are present, the risk of dental complications is high even after a relatively short history of vomiting [110] and dental reviews are recommended. Common gastrointestinal disturbances include gastric pain, prolonged gastric emptying and constipation [108], all of which can perpetuate the desire to continue restricting.

The presence of medical complications may or may not lead to hospital admission. As noted earlier, significant contextual factors, availability and regional treatment philosophies influence admission decisions [111] both within and between countries. When acute medical presentations occur, particular care is needed to avoid the refeeding syndrome and other treatment-related complications. Medical symptoms may be alarming and prompt consideration of urgent steps, but cautious use of otherwise considered routine interventions (for example, intravenous fluids) is required in order to avoid catastrophic consequences [112]. A small number of patients will require an explicit medical admission.

### Compulsory assessments

On occasions, preliminary assessments occur under duress after formal steps have been taken to initiate the assessment. Engagement of a reluctant patient who may require persuasion, formal or otherwise, to accept urgent investigations or treatment requires skill and tact. The legal options and legislative pathways for compulsory assessment vary across countries, but the principles are relatively universal and well discussed in the literature [113,114]. Legal coercion is required relatively infrequently [115]. Importantly, patient perceptions of coercion, with or without the use of formal compulsion, occur along a continuum [116] with many later describing formal treatment orders as helpful. Much can be done to mitigate distress when a compassionate and trusting approach is taken [117]. Decision-making must consider patient capacity to make decisions, the urgency of presenting symptoms and the outcomes where compulsory steps have been previously utilised to detain the patient.

### Synthesis and formulation of assessment information

A comprehensive assessment interview is likely to take several hours, and where clinically indicated, may require several appointments. Once sufficient and necessary information has been gathered, the next step is to synthesise this with other findings from other standard assessment steps such as history taking and mental status examination.

A case formulation approach is recommended to develop “*a more complete picture of the patient than can be associated with a diagnosis*” [[118], page 1] incorporating all presenting issues, past treatment outcomes and patient perspectives. It involves assimilating information from multiple sources and using clinical judgement to weigh up both converging and conflicting data. A case formulation approach is also conducive to building a therapeutic alliance which is vital in psychotherapeutic treatments both generally and specifically in regarding to AN [119].

The formulation may include tentative hypotheses about predisposing, precipitating and maintaining factors. Families commonly ask about causal factors fearing that assessment findings will implicate them in some way. Pressed for answers from distressed families (and patients themselves), clinicians may be tempted to be drawn on predisposing factors. However causes of AN are far from understood. Tentative hypotheses are best, and better framed as general risk factors in the interim. Given the typically prolonged time between onset and assessment combined with difficulties establishing age of onset, articulating more immediate precipitating factors is equally fraught. It will be more possible to draw conclusions about maintaining factors due to the extensive knowledge about reinforcement schedules and behavioural traps in the lived experience of AN.

In a large multidisciplinary team, varying perspectives and formulations may emerge and this can be clinically useful. However such differences should never divert from the importance of all team members uniting behind any arising triage or treatment plan. The end result of the case formulation should be a set of working hypotheses articulated into a coordinated care plan followed by all team members. This care plan should be both detailed and broad covering immediate treatment needs and priorities for further investigation.

## Conclusion

This overview in limited in its ability to fully discuss all relevant factors in assessment; a decision was made to include issues that would be pertinent to a wide audience. Likewise, the scope of this overview precludes discussion of the steps instigating treatment following from assessment, although it follows that management of acute physical and psychiatric problems naturally take priority.

Experienced clinicians know that the process of assessment is an on-going one, encompassing a need to repeat and monitor medical investigations and revise psychological formulations as more information comes to light, symptoms worsen or improve and priorities shift. A preliminary assessment should never attempt to reach firm opinions about cause although formulations relating to factors maintaining the disorder may be more apparent.

In conclusion, assessments require a systematic, rigorous, and empathic approach that strikes an important balance between carefully listening to patient needs and dilemmas, while remaining firm and focused about the need for treatment. Even difficult assessment interviews conducted under duress should leave the patient with a sense of dignity and that they have been heard. The assessment interaction should be seen as an important therapeutic interaction to begin building a therapeutic alliance, balancing both an atmosphere of acceptance [120] yet firmness without being over-confident or paternalistic. The most important assessment tool is the clinician and the knowledge and stance they take into the interpersonal interaction. An assessment will always be a deeply complex interpersonal interaction requiring a careful clinical interview, and one based on a sound understanding of the symptoms and issues faced by the patient.

On-going efforts are required to address substantive knowledge gaps and to encourage the uptake of evidenced-based approaches where such evidence is assessed as strong. As noted recently, “*the bond that tethers our patient to our treatment is a fragile one*” [[121], page 178]. At times this could be rephrased to say that the bond that tethers assessing clinicians to the evidence is equally fragile. There is a universal problem transferring research evidence into clinical practice, and this overview aims to reduce that ‘leakage’ [122] between research and practice.

While not above criticism themselves, a number of practice guidelines are available that outline in some detail the required and desired assessment investigations, and at least draw clinicians’ attention to the quality and limitations of evidence. These include the Australasian [123] (currently undergoing review), North American [124], and the British [101] guidelines. The latter of these (and the most recently published) appeared just under a decade ago, but a recent review of the literature indicates that these guidelines are as relevant today as they were in 2004, (http://www.nice.org.uk/nicemedia/live/10932/55781/55781.pdf, downloaded 17 Sept 2012).

## Competing interests

Both authors declare that they have no competing interest.

## Authors’ contributions

LS carried out the literature review and drafted the early version. SM revised initial manuscript drafts and critically commented on the intellectual content. Both authors read and approved the final manuscript.
